# Androgen and Estrogen Receptor Expression in Different Types of Perianal Gland Tumors in Male Dogs

**DOI:** 10.3390/ani11030875

**Published:** 2021-03-19

**Authors:** Adam Brodzki, Wojciech Łopuszyński, Yolanda Millan, Marcin R. Tatara, Piotr Brodzki, Katarzyna Kulpa, Natalia Minakow

**Affiliations:** 1 Department and Clinic of Animal Surgery, Faculty of Veterinary Medicine, University of Life Sciences in Lublin, 20-612 Lublin, Poland; brodzkiadam@op.pl (A.B.); kulpa.kasia@gmail.com (K.K.); minakownatalia@gmail.com (N.M.); 2 Sub-Department of Pathomorphology and Forensic Veterinary Medicine, Department and Clinic of Animal Internal Diseases, University of Life Sciences in Lublin, 20-612 Lublin, Poland; 3 Department of Anatomy and Comparative Pathology, Córdoba University, 14071 Córdoba, Spain; an2mirum@uco.es; 4 Department of Animal Physiology, Faculty of Veterinary Medicine, University of Life Sciences in Lublin, 20-950 Lublin, Poland; matatar99@gazeta.pl; 5 Department of Andrology and Biotechnology of Reproduction, Faculty of Veterinary Medicine, University of Life Sciences in Lublin, 20-612 Lublin, Poland; wetdoc@interia.pl

**Keywords:** perianal gland tumors, male dogs, estrogen receptor, androgen receptor, immunohistochemistry

## Abstract

**Simple Summary:**

The immunohistochemical evaluation of the steroid hormone receptors expression in neoplastic cells is a widely used diagnostic method in human medicine especially for the selection of appropriate therapy in case of breast cancers. The authors made an attempt to demonstrate prognostic and predictive value of determination of sex hormone receptors in canine perianal gland tumors. Perianal adenomas, epitheliomas and carcinomas occur mainly in older male dogs. Although surgery is the most commonly used procedure, the risk of complications related to general anesthesia and the anatomical location of the tumor adjacent to the anus makes a dilemma. The performed studies indicate that perianal gland tumors in dogs with a high expression of androgen and estrogen receptors may show a potentially greater sensitivity to hormone therapy in contrast to tumors showing low or no expression. Moreover, it was noticed that the expression of the receptors was significantly lower in neoplasms with high histological malignancy grade compared to benign tumors. Diversified expression of androgen and estrogen receptors depending on the histological type can be used for further clinical studies aimed at developing diagnostic and prognostic schemes providing the selection of therapy in the case of perianal gland tumors in dogs.

**Abstract:**

Perianal gland tumors are modified sebaceous glands present in the skin of the perianal region in the dog. Hormonal stimulation may induce hyperplasia of the perianal glands or their neoplastic progression. The presence of androgen (AR) and estrogen (ER) receptors have been demonstrated both in normal perianal glands as well as in perianal tumors. The aim of the study was an immunohistochemical assessment of the expression of estrogen and androgen receptors in perianal gland tumors in dogs as an applicatory marker for antihormonal treatment. Biopsy samples of perianal masses were collected from 41 male dogs. A histopathological examination revealed 24 adenomas, 12 epitheliomas and five carcinomas. The immunohistochemical staining showed a mainly nuclear expression of AR and ER in the neoplastic cells. Both the androgen and estrogen receptors were expressed in adenoma, epithelioma and carcinoma cases; however, the highest expression of the receptors was stated in the adenoma and epithelioma. In the case of the carcinoma, the expression of sex hormone receptors was very weak. The differences of the number of cells expressing AR and ER as well as the observed differentiated intensity of staining in the studies demonstrated that the determination of the expression of the sex hormone receptors may be useful to elaborate a diagnostic and therapeutic algorithm.

## 1. Introduction

Perianal gland tumors rank third among all types of male dog tumors and their pharmacological treatment is a challenge for modern oncology [1,2]. Perianal glands belong to the modified sebaceous glands present in dogs in the skin of the perianal region, perineum, prepuce, medial femoral region, base of tail and also other body regions occasionally. Due to their morphological similarity to the arrangement of hepatocytes they are also called hepatoid glands. Androgen hormones stimulate the development of perianal glands [3,4]. Hormonal stimulation in adult and aged male dogs may induce hyperplasia of the perianal glands or their neoplastic transformation [5]. Contrary to males, a gradual regression of the perianal glands is observed in aged intact females that is potentially associated with the biological activity of estrogens. However, in spayed females perianal adenomas almost exclusively occur due to the fact that estrogens do not inhibit tumor growth [6,7]. A clear etiology of perianal gland tumors has not been completely recognized yet; however, hormonal factors associated with the male sex were postulated as important causative factors responsible for a neoplastic progression [8]. Perianal gland tumors occur mainly in male dogs that are not subjected to orchidectomy while their occurrence in females is very rare especially in spayed bitches [9]. According to previous reports, a long-term biological exposure to testosterone in male dogs is considered to result in an increased risk of the development of perianal gland tumors while in spayed bitches the endogenous androgens secreted by the adrenal glands may be decisive. Hormonal interactions and their involvement in the etiology of perianal gland tumors have been confirmed in clinical studies. Significantly better therapeutic effects of the simultaneous surgical removal of neoplastic lesions and orchidectomy in male dogs rather than a single surgical removal of a tumor have been documented [10]. The presence of steroid hormone receptors was confirmed both in healthy glands and neoplastic lesions, indicating the potential therapeutic application of a pharmacological treatment [11]. Nowadays, it is well known that both the normal perianal (hepatoid) gland cells and neoplastic cells of the perianal tumors show the presence of androgen and estrogen receptors. Estrogens and androgens stimulate cellular neoplastic proliferation and induce agonistic effects by the activation of receptors. Thus, pilot approaches to a conservative treatment of a few neoplastic diseases have been executed using drugs with an antiandrogen and antiestrogen activity. It has been suggested that a conservative pharmacological treatment may be effective also in the case of perianal gland tumors [11].

The aim of the study was the immunohistochemical determination of the expression of androgen and estrogen receptors in the neoplastic cells of perianal gland tumors in dogs and the contents of its potential application as a marker for an antihormonal pharmacological treatment.

## 2. Materials and Methods

### 2.1. Study Material

A laboratory analysis was performed using tumor tissue samples of perianal glands collected during a punch needle biopsy in dogs handled by the Department and Clinic of Animal Surgery of the University of Life Sciences in Lublin. A skin punch with a 6 mm diameter was used for the biopsy of the tumors used in histopathological studies. The collected tissue samples were fixed in 10% formalin (pH = 7.2) for 24 h and processed for 24 h using a tissue processor (Leica TP-20, Leica Biosystems, Nussloch, Germany) in an ascending ethanol concentration solution array of acetone and xylene and embedded in paraffin. Trick tissue slices of 4 μm were cut using a sledge microtome (Leica SR-200, Leica Biosystems, Nussloch, Germany) and placed on glass slides. Histopathological sections stained with hematoxylin and eosin (HE) were analyzed using a light microscope (Nikon Eclipse E-600, Nikon Instruments, Tokyo, Japan). The microscopic assessment of the tissue sections was based on the histological classification of epithelial tumors proposed by Armed Forces Institute of Pathology/Word Health Organization (AFIP/WHO) [12].

### 2.2. Immunohistochemistry

Sections of tissue slices used for immunohistochemical staining were placed on Superfrost^TM^ silane-covered slides (Menzel–Glaser, Germany) and put to the heater at 56 °C for 12 h. After the incubation, the samples were deparaffinized in xylene and rehydrated through a descending ethanol solution and distilled water array. An antigen unmasking procedure was applied before the immunohistochemical reaction. Histological preparations for the determination of the expression of estrogen receptors (ER) and androgen (AR) receptors were put into a citrate buffer (pH = 6.0) and unmasked in a water bath at a temperature of 98 °C for 30 min. After the unmasking procedure, the histological section was left in baths to reach room temperature. An antigen-antibody complex system containing secondary biotin-conjugated antibodies toward mouse or rabbit primary monoclonal or polyclonal antibodies LSAB plus HRP (Dako, Golstrup, Denmark) was used for the immunohistochemical detection of ER and AR [13]. The diluted 1:50 primary mouse monoclonal antibodies against the estrogen receptor (anti-Estrogen Receptor clone TE111.5D11; Acris Antibodies, Herford, Germany) and against the androgen receptor (anti-Androgen Receptor Ab-1 clone AR 441; Thermo Scientific, Fremont, USA) were used in the immunohistochemical evaluation. The sections with primary antibodies were applied for overnight incubation at +4 °C. Streptavidin conjugated to horseradish peroxidase served as the enzyme detecting area of the reaction while the reagent (chromogene) 3,3′-diaminobenzidine tetrahydrochloride (DAB; SK-4100, Vector Laboratories, Peterborough, UK) induced a color reaction. The solution for the chromogenic reaction was prepared 10 min prior to the application and contained 10 mg DAB in 10 mL 0.05 M Tris-HCl (pH = 7.4) and 3% H_2_O_2_ in an amount of 100 μL. A double control system of the immunohistochemical reactions was applied. The negative control was based on the replacement of the primary antibody incubation with the corresponding non-immunized commercial serum containing IgG, while the positive control was executed using an incubation of the target tissues showing proven positive reactions of the antibodies with the antigen. The samples of a uterus obtained from a healthy bitch and human prostate gland samples were used as the target tissues in the study.

### 2.3. ER and AR Expression Evaluation

A quantitative evaluation of the expression of ER and AR was performed using a computer-assisted microscopic image analysis system consisting of a light microscope (Nikon Eclipse E-600, Nikon Instruments, Tokyo, Japan) coupled with a digital camera (Nikon DS-Fi1, Nikon Instruments, Tokyo, Japan) and PC computer operated software for image analysis (NIS-Elements BR-2.20, Laboratory Imaging, Praha, Czech Republic). In the first step of the microscopic evaluation using a 10-time objective magnification, the histological fields of view showing a characteristic cellular structure and the highest nuclear expression of the receptors for several types of the evaluated tissue were selected for analysis. Microscopic images were analyzed after image filter adjustment and determination of the cut-off threshold in correspondence with the positive control tissues [14,15]. A semi-quantitative method of the estrogen and androgen expression evaluation was applied in the study using the 4-level proportional score (PS) scale (0–3) based on the percentage of cells showing a staining reaction and the intensity of staining score (ISS) (score scale 1–3) [16]. A lack of the presence of the cells showing a positive reaction for staining corresponded with the PS score 0 while 10–30% of cells showing a positive reaction to staining corresponded with the PS score 1. The presence of 31–60% cells showing a positive reaction for staining corresponded to the PS score 2 while the PS score 3 reflected the number of cells with a positive reaction for staining over 60%. A poor (weak) intensity of staining corresponded to the ISS score 1, a moderate (medium) intensity of staining corresponded to the score 2 and a high (strong) intensity of staining corresponded to the score 3. The total score (TS) was calculated as the arithmetic sum of the proportional score (reflecting the number of cells with an expression of the receptor) and the intensity of staining score (TS = PS + ISS). Total score values were obtained separately for androgen and estrogen receptors for both the basaloid and the hepatoid cells. Finally, a total expression score (TES) was calculated separately for androgen and estrogen receptors as the arithmetic sum of the total scores for basaloid and hepatoid cells in different types of perianal gland tumors. The PC computer operated software for image analysis was used for digital image archive collection.

### 2.4. Statistical Analysis

The results obtained in the study were presented as mean values and standard deviations (±SD). A statistical comparison of the data was performed using the non-parametric Wilcoxon matched-pairs signed rank test and the non-parametric Kruskal–Wallis test for multiple comparisons. The level of statistical significance was set at *p* ≤ 0.05 for all comparisons.

## 3. Results

### 3.1. Dog Population and Histopathology

**Figure 1:**
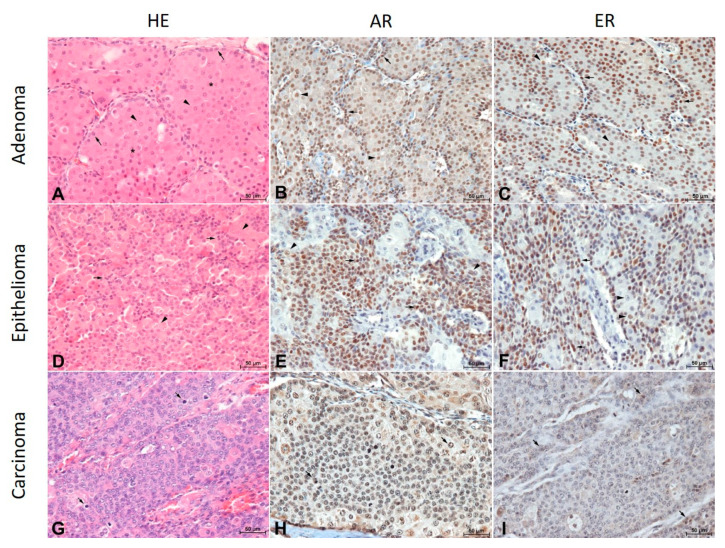
(**A**). Perianal gland adenoma composed of uniform broad trabeculae and islands (asterisks) of polygonal cells (arrows) surrounded by a single layer of attenuated basaloid reserve cells (arrowheads). HE, magnification × 200, bar = 50 µm. (**B**). Intense nuclear reaction to the androgen receptor in basaloid cells (arrows) and slightly weaker reaction in hepatoid cells (arrowheads) in a perianal gland adenoma. Indirect immunohistochemistry. Mayer’s hematoxylin counterstain, magnification × 200, bar = 50 µm. (**C**). A strong reaction to the estrogen receptor in basaloid cells (arrows) and a moderate reaction in hepatoid cells (arrowheads) in a perianal gland adenoma. Indirect immunohistochemistry. Mayer’s hematoxylin counterstain, magnification × 200, bar = 50 µm. (**D**). Proliferating basaloid cells (arrows) in the vicinity of less numerous hepatoid cells (arrowheads) in a perianal gland epithelioma. Indirect immunohistochemistry. Mayer’s hematoxylin counterstain, magnification × 200, bar = 50 µm. (**E**). The diversity of an androgen receptor expression in perianal gland epithelioma cells. Basaloid tumor cells were highly expressed (arrows) while hepatoid cells were predominantly low expressed (arrowheads). Indirect immunohistochemistry. Mayer’s hematoxylin counterstain, magnification × 200, bar = 50 µm. (**F**). A strong nuclear estrogen receptor expression in proliferating basaloid cells (arrows) and moderate to low expression in hepatoid cells (arrowheads) in a perianal gland epithelioma. Indirect immunohistochemistry. Mayer’s hematoxylin counterstain, magnification × 200, bar = 50 µm. (**G**). Perianal gland carcinoma. Undifferentiated, pleomorphic cells with numerous hyperchromatic cell nuclei and mitotic figures (arrows). HE, magnification × 200, bar = 50 µm. (**H**). Low-differentiated cells of a perianal gland carcinoma, some with mitotic figures (arrows) with a low expression of the androgen receptor. Indirect immunohistochemistry. Mayer’s hematoxylin counterstain, magnification × 200, bar = 50 µm. (**I**). A low expression of the estrogen receptor in perianal gland carcinoma cells (arrows). Indirect immunohistochemistry. Mayer’s hematoxylin counterstain, magnification × 200, bar = 50 µm.

Forty one male intact dogs with perianal gland tumors were included in the study. The mean age of the dogs was 6.8 years old (range 4–17). Purebred dogs (25; 61%) predominated in the research population. Among the purebred dogs, small breeds dominated in the following order: Cocker Spaniels (6; 15%), Wire-Haired Fox Terriers (3; 7%), Dachshunds (2; 5%) and Miniature Pinscher (2; 5%). Among the large breed dogs, the German Shepherd was the most numerous (2; 5%). The remaining breeds were represented by one individual. Among the group of 41 perianal tumors, 24 cases (58.5%) were recognized as an adenoma, 12 cases (29.3%) were recognized as an epithelioma and five cases (12.2%) were classified as a carcinoma. The similarity of the microscopic features of the hyperplasia and hepatoid adenoma resulted in a common classification of both lesions to the adenoma group in our study. The immunohistochemical visualization revealed a mainly nuclear expression of AR and ER in the neoplastic cells while the cytoplasmic expression of ER was occasional and very weak (Figure 1).

### 3.2. Expression of Androgen Receptors in Basaloid and Hepatoid Cells in Different Types of Neoplastic Lesions

**Figure 2:**
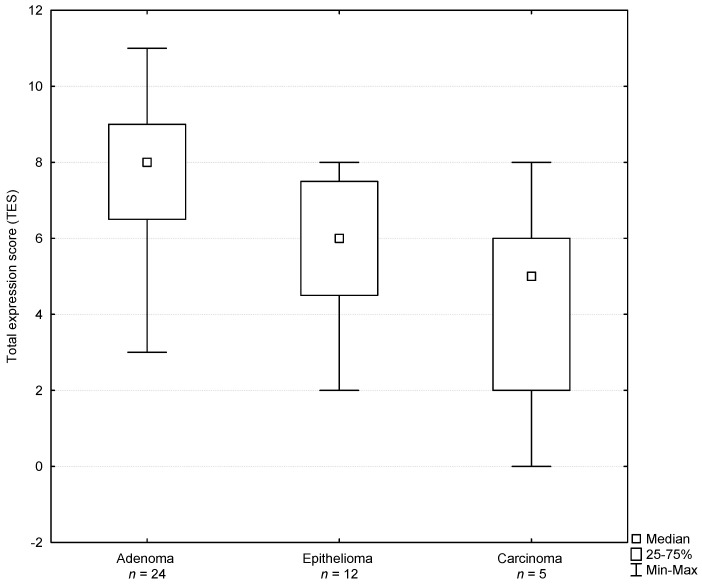
Total expression score determined for androgen receptors in perianal gland tumors of male dogs.

The results of the statistical analysis of the androgen receptor expression in the studied tumors are presented in Table 1. A proportional score of basaloid cells showing the expression of androgen receptors reached 2.33 ± 0.56 in the adenoma group, 2.08 ± 0.66 in the epithelioma group and 1.40 ± 0.89 in the carcinoma group and the differences between the groups were not significant (*p* > 0.05). The intensity of the staining score of androgen receptors in basaloid cells reached 2.00 ± 0.66 in the adenoma group, 1.42 ± 0.51 in the epithelioma group and 1.00 ± 0.70 in the carcinoma group and the differences were significant between the adenoma and carcinoma groups (*p* = 0.04). A total score value for androgen receptors in basaloid cells reached 4.33 ± 1.00 in the adenoma group, 3.50 ± 0.67 in the epithelioma group and 2.40 ± 1.51 in the carcinoma group and the differences were significant between the adenoma and carcinoma groups (*p* = 0.01). A proportional score of hepatoid cells showing the expression of androgen receptors reached 1.67 ± 0.91 in the adenoma group, 1.42 ± 1.08 in the epithelioma group and 1.00 ± 1.00 in the carcinoma group and the differences between the groups were not significant (*p* > 0.05). The intensity of the staining score of androgen receptors in hepatoid cells reached 1.50 ± 0.93 in the adenoma group, 0.83 ± 0.58 in the epithelioma group and 0.80 ± 0.84 in the carcinoma group and the differences between the groups were not significant (*p* > 0.05). A total score value for androgen receptors in hepatoid cells reached 3.16 ± 1.61 in the adenoma group, 2.25 ± 1.54 in the epithelioma group and 1.80 ± 1.79 in the carcinoma group and the differences between the groups were not significant (*p* > 0.05). A total expression score for androgen receptors (both in basaloid and hepatoid cells) reached 7.50 ± 2.39 in the adenoma group, 5.75 ± 1.86 in the epithelioma group and 4.20 ± 3.19 in the carcinoma group and the differences were significant between the adenoma and carcinoma groups (*p* = 0.04) and between the adenoma and epithelioma groups (*p* = 0.05; Table 1 and Figure 2). Basaloid cells expressing androgen receptors in the adenoma group and in the epithelioma group were significantly more numerous than hepatoid cells (both *p* < 0.05). Basaloid cells in the adenoma group showed a significantly higher ISS of androgen receptors than hepatoid cells (*p* = 0.007) and a similar tendency was observed in the epithelioma group (*p* = 0.06). The total score for androgen receptors in the adenoma group and in the epithelioma group reached significantly higher values in basaloid cells than in hepatoid cells (*p* ≤ 0.01).

### 3.3. Expression of Estrogen Receptors in Basaloid and Hepatoid Cells in Different Types of Neoplastic Lesions

**Figure 3:**
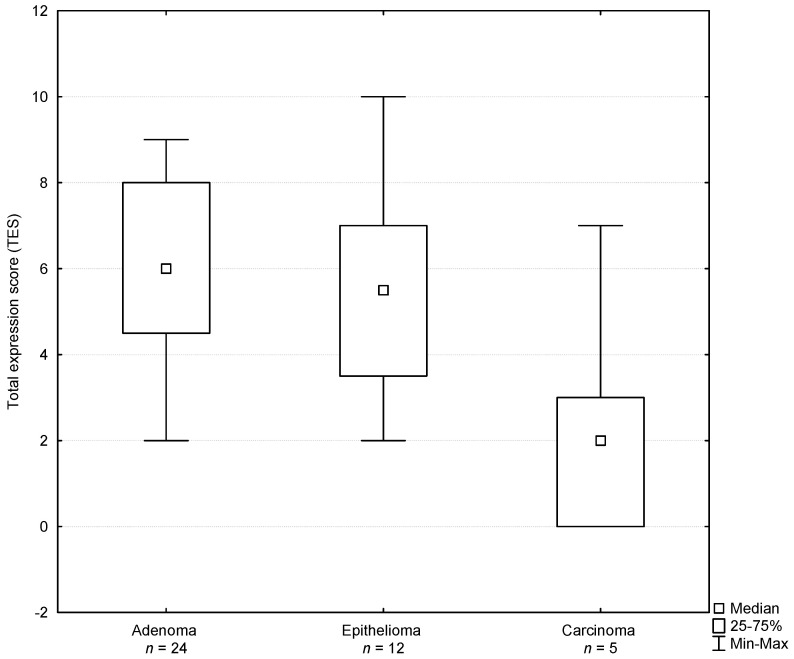
Total expression score determined for estrogen receptors in perianal gland tumors of male dogs.

The results of a statistical analysis of the estrogen receptor expression are presented in Table 1. A proportional score of basaloid cells showing the expression of estrogen receptors reached 2.12 ± 0.80 in the adenoma group, 1.92 ± 0.67 in the epithelioma group and 1.20 ± 1.30 in the carcinoma group and the differences between the groups were not significant (*p* > 0.05). The intensity of the staining of estrogen receptors in basaloid cells reached 2.17 ± 0.70 in the adenoma group, 1.67 ± 0.78 in the epithelioma group and 0.80 ± 0.84 in the carcinoma group and the differences were significant between the adenoma and carcinoma groups (*p* = 0.01). A total score value for estrogen receptors in basaloid cells reached 4.29 ± 1.20 in the adenoma group, 3.58 ± 1.38 in the epithelioma group and 2.00 ± 2.12 in the carcinoma group and the differences were significant between the adenoma and carcinoma groups (*p* = 0.04). A proportional score of hepatoid cells showing the expression of estrogen receptors reached 0.83 ± 0.70 in the adenoma group, 0.92 ± 0.79 in the epithelioma group and 0.20 ± 0.45 in the carcinoma group and the differences between the groups were not significant (*p* > 0.05). The intensity of the staining score of estrogen receptors in hepatoid cells reached 0.83 ± 0.70 in the adenoma group, 0.92 ± 0.80 in the epithelioma group and 0.20 ± 0.45 in the carcinoma group and the differences between the groups were not significant (*p* > 0.05). A total score value for estrogen receptors in hepatoid cells reached 1.67 ± 1.31 in the adenoma group, 1.83 ± 1.53 in the epithelioma group and 0.40 ± 0.89 in the carcinoma group and the differences between the groups were not significant (*p* > 0.05). A total expression score for estrogen receptors both in basaloid and hepatoid cells reached 5.96 ± 2.14 in the adenoma group, 5.42 ± 2.50 in the epithelioma group and 2.40 ± 2.88 in the carcinoma group and the differences were significant between the adenoma and carcinoma groups (*p* = 0.04; Table 1 and Figure 3). Basaloid cells in the adenoma group and in the epithelioma group showed a significantly higher ISS of estrogen receptors than hepatoid cells (*p* ≤ 0.01). The total score for estrogen receptors in the adenoma group and in the epithelioma group reached significantly higher values in basaloid cells than in hepatoid cells (both *p* < 0.01).

## 4. Discussion

The results of previous studies showed that perianal gland tumors occur most frequently in non-orchidectomized male dogs older than six years while spayed bitches suffer from perianal gland tumors occasionally [17,18]. In our study, tissue samples were collected only from male dogs suffering from perianal gland tumors due to the occurrence of these tumors almost exclusively in male patients in the dog population. In previous reports, the cellular expression of sex hormone receptors was documented in tissues undergoing a neoplastic transformation [11,19]. The results of the current studies showed mainly the expression of ER in the cellular nuclei of the neoplastic cells while a cytoplasmic expression of ER was occasional and weak. The androgen receptor expression was also stated mainly in cellular nuclei. Both the estrogen and androgen receptors were expressed in the adenoma, epithelioma and carcinoma cases; however, the highest total expression scores (TES) were stated in the adenoma and then in the epithelioma representing a low grade malignant neoplasm. In the case of a carcinoma of the perianal gland tumor, the expression of sex hormone receptors was very weak. These findings were in accordance with the previous report by Pisani (2006) where AR expression was found in adenomas, epitheliomas and carcinomas [11]. A cellular expression of AR in the investigated group of tumors was similar in all types of the neoplastic lesions. Contrary to the current study, the previous report did not show statistically significant differences of the cellular expression of AR comparing benign and malignant neoplastic lesions. The investigation on 19 dogs suffering from perianal gland tumors showed a moderate neoplastic transformation in nine cases (47.4%), seven cases (36.8%) were well differentiated and poor neoplastic differentiation was found in three cases (15.8%). Statistically significant differences were not found comparing the percentage of cells showing an AR expression in poor, moderate and well differentiated neoplastic changes [11]. Contrary to the previous report, a significantly higher expression of AR receptors was found in the adenoma than in the carcinoma groups in the current study. The results of the current study, showing a lower expression of ER in carcinomas than in adenomas, were in accordance with the previous analyses of the expression of ER and progesterone receptors (PR) in neoplastic and normal tissue samples collected from male and female dogs [18]. In the previous studies, the expression of PR was higher in adenomas than in carcinomas. The expression of progesterone receptors was higher in adenomas also when compared with healthy perianal gland tissue. In both sexes of dogs, the expression of ER and PR in carcinomas was significantly lower than in adenomas [18]. In addition to the assessment of the expression of receptors in the current study, the intensity of the staining score was also determined. The differences of the ISS between basaloid and hepatoid cells were similar in the case of AR and ER. Both AR and ER showed significantly higher ISS in basaloid cells than in hepatoid cells. The intensity of the staining score of the receptors, similar to their total expression score, was higher in the adenoma and the epithelioma groups than in the carcinoma group. It is interesting that in studies on neoplastic lesions of the prostate gland in humans by Husain (2016), completely different dependences were found [19]. Both the expression and intensity of staining were compared in benign prostatic hyperplasia and prostatic intraepithelial neoplasia and adenocarcinoma. A significantly higher intensity of staining of AR was found in the case of prostate gland adenocarcinoma than in benign prostate gland hyperplasia. Thus, the intensity of the staining determination of ER and AR in prostate gland neoplastic lesions may be considered as a potential prognostic indicator of the disease progression [19]. It cannot to be excluded that in dogs suffering from perianal gland tumors the intensity of the staining score of AR and ER may be potentially used as a prognostic factor even it shows different dependencies than in the case of pathological prostate gland changes. However, a standardization procedure of the methodological approach of AR and ER staining is required for such purposes. The previous studies also concerned the expression of sex hormone receptors in an endometrial carcinoma (ECA) in women [20]. Even though an ECA often shows a hormonal etiology (and endocrine-based therapy would be possible) the evaluation of hormone receptors is not often determined. Although the role of AR in an ECA was not explained completely, it was shown that androgens had antiproliferative effects on an unchanged endometrium and may act similarly to progesterone for the inhibition of estrogen-dependent neoplastic processes. Thus, in a few cases of an ECA, androgen antagonistic therapy may bring positive therapeutic effects. In previous histological studies on different types of ECA, the comparison of the expression of AR, ER and PR was performed. The expression of androgen receptors was stated in 54% of the investigated samples (27/50 cases) of an ECA that contributed to low grade endometroid carcinomas in 60%, high grade endometroid carcinomas in 70%, serous carcinomas in 70%, carcinosarcomas in 50% and clear cell carcinomas in 20%. A noticeably higher AR expression was mainly stated in the subgroup of serous carcinomas, reaching 50%. Moreover, it was concluded that the serous carcinoma subgroup was characterized by a high AR expression in half of the investigated cases and a subset of serous carcinomas and carcinosarcomas showed some AR staining appearance with a simultaneous lack of ER staining, suggesting the possibility of the application of antiandrogen therapy in these cases [20]. The expression of estrogen receptors in neoplastic cells plays a crucial role in cases of breast cancer in women. The differentiation of the breast cancer type in human medicine is based on the presence of ER. Thus, an ER-positive breast cancer showing ER expression and an ER-negative breast cancer showing a lack of ER expression may be distinguished [21]. Breast cancer differentiation in accordance with ER expression serves both as a prognostic indicator and an appropriate therapeutic approach determinant. The therapeutic prognosis is better in breast cancer cases showing ER expression [22] but ER-negative cases are more aggressive and induce neoplastic metastases more frequently [23]. It is important that breast cancer cases showing ER expression may be subjected to antihormonal treatment and the ER expression determination indicates a proper therapeutic approach using antiestrogens [21]. The immunohistochemical determination of the ER expression in breast cancers in women is routinely used as a prognostic indicator and serves as a predictor for the therapy [24]. Based on the presented results it can be assumed that this method could also be used in the selection of patient to pharmacological treatment in the case of perianal gland tumors in dogs [2]. There are no diagnostic methods that would enable the selection of patients for such treatment therefore an immunohistochemical assessment of the expression of AR and ER receptors in perianal gland tumors in dogs could be used as one of the predictors for therapeutic purposes. The limitations, however, result from the fact that immunohistochemistry requires specialized equipment, an appropriate interpretation of results and in particular, the standardization of the method so that it can be widely used in veterinary practice.

Mammary gland tumors in female dogs are considered as a potential experimental model imitating hormone-dependent breast cancer in women [25]. This hypothesis was confirmed in the immunohistochemical studies on the expression of ER-alpha in neoplastic mammary gland tissues [26]. In bitches subjected to a clinical examination and retrospective analysis of their reproduction history, the surgical removal of the neoplastic changes was followed for 18 months including clinical examinations every 3–4 months. It revealed that malignant mammary gland tumor tissues in bitches with an imaginary pregnancy history showed a significantly higher expression of ER-alpha. Immunoexpression of ER-alpha was lower progressively in accordance with an increasing tumor size and an appearance of skin ulceration. A low expression of ER-alpha was associated with neoplastic metastases to lymphatic nodes. Furthermore, primarily malignant tumors showing a low ER-alpha expression were associated frequently with a later appearance of metastases. Thus, the previous studies in bitches suffering from mammary gland tumors showed that the immunohistochemical determination of ER-alpha expression may be considered as a relatively simple method with a prognostic clinical value serving also for the proper selection of a hormonal/antihormonal therapy application in the patient [26]. The results obtained in the current study, which showed a lower ER expression in a malignant neoplasm (carcinoma) than in a benign adenoma and epithelioma, corresponded with previous findings in bitches with mammary gland tumors even though a different biological material (tumor type) was evaluated [25,26]. The potential predictive value of immunohistochemical PR receptor determination in canine mammary carcinomas was demonstrated in studies of the PR antagonist aglepristone. A neoadjuvant aglepristone treatment increased the disease-free period in animals with tumors expressing the PR receptor [27]. Experimental studies on receptor expression in neoplastic tissues were also performed in animal species other than dogs. In female cats, the expression of ER and PR in mammary gland tumors was evaluated [28]. It was shown that the ER expression was significantly higher in healthy tissues and in adenosis than in neoplastic lesions. The progesterone receptor expression was elevated in fibroadenomatous changes and in in situ carcinomas and decreased in invasive carcinomas. However, the correlation of ER and PR expression levels in invasive carcinomas with histological parameters and animal survivability was not confirmed even though tumors showing a lack of ER expression were related with a poor prognosis [28]. Based on an experienced efficiency of the antihormonal therapy of neoplastic lesions showing the expression of sex hormone receptors and the described method of AR/ER expression evaluation in canine perianal gland tumors it is advisable to undertake pathological and clinical studies enabling the implementation of diagnostic and therapeutic algorithms based on the level of hormone expression [2].

## 5. Conclusions

The obtained results as well as experience from human medicine led us to the conclusion that the sensitivity of neoplastic transformed cells to antihormonal therapy may be predicted based on the determination of the expression of the sex hormone receptors resulting from a quantitative determination of the expression of receptors (PS) and their intensity of staining (ISS). Thus, both these parameters as well as their arithmetic summation expressed as total expression score value (TES) may be useful to verify antihormonal treatment convenience in each individual case. Nevertheless, in order to be able to recommend the assessment of AR and ER expression according to the method used as predictors for antihormonal therapy in the case of canine perianal gland tumors in clinical practice, further clinical studies are necessary to establish the relationship between the level of AR and ER expression and the effect of the therapy.

## Figures and Tables

**Table 1:** Proportional score, intensity of staining score, total score and total expression score values in perianal gland tumors of male dogs.

Receptor Type	Type of Cells	Score	Adenoma *n* = 24 Mean Value ± SD	Epithelioma *n* = 12 Mean Value ± SD	Carcinoma *n* = 5 Mean Value ± SD
AR	Basaloid cells	PS	2.33 ± 0.56	2.08 ± 0.66	1.40 ± 0.89
		ISS	2.00 ± 0.66 ^a^	1.42 ± 0.51 ^ab^	1.00 ± 0.70 ^b^
		TS	4.33 ± 1.00 ^a^	3.50 ± 0.67 ^ab^	2.40 ± 1.51 ^b^
	Hepatoid cells	PS	1.67 ± 0.91	1.42 ± 1.08	1.00 ± 1.00
		ISS	1.50 ± 0.93	0.83 ± 0.58	0.80 ± 0.84
		TS	3.16 ± 1.61	2.25 ± 1.54	1.80 ± 1.79
	TES		7.50 ± 2.39 ^a^	5.75 ± 1.86 ^b^	4.20 ± 3.19 ^b^
ER	Basaloid cells	PS	2.12 ± 0.80	1.92 ± 0.67	1.20 ± 1.30
		ISS	2.17 ± 0.70 ^a^	1.67 ± 0.78 ^ab^	0.80 ± 0.84 ^b^
		TS	4.29 ± 1.20 ^a^	3.58 ± 1.38 ^ab^	2.00 ± 2.12 ^b^
	Hepatoid cells	PS	0.83 ± 0.70	0.92 ± 0.79	0.20 ± 0.45
		ISS	0.83 ± 0.70	0.92 ± 0.80	0.20 ± 0.45
		TS	1.67 ± 1.31	1.83 ± 1.53	0.40 ± 0.89
	TES		5.96 ± 2.14 ^a^	5.42 ± 2.50 ^ab^	2.40 ± 2.88 ^b^

^a,b^ Means that share different upper superscript letters differed significantly for *p* ≤ 0.05. AR: androgen receptors. ER: estrogen receptors. PS: proportional score. ISS: intensity of staining score. TS: total score. TES: total expression score.

## Data Availability

The datasets used and analyzed in the current study are available from the corresponding authors on reasonable request.
